# Does gestational vitamin D attenuate the negative effects of prenatal depression on offspring emotional and behavioral problems? Findings in the ECHO cohort

**DOI:** 10.1017/S0033291726104917

**Published:** 2026-06-16

**Authors:** Alison E. Hipwell, Irene Tung, Meredith Palmore, Melissa M. Melough, Lisa M. Bodnar, Lisa A. Croen, Ashley V. Hill, Traci A. Bekelman, Patricia A. Brennan, Kecia N. Carroll, Rebecca J. Schmidt, Emily Zimmerman, Monica McGrath, ECHO Cohort Consortium

**Affiliations:** 1 Department of Psychiatry, University of Pittsburgh, Pittsburgh, USA; 2 Department of Psychology, California State University, Dominguez Hills, Carson, USA; 3 Department of Epidemiology, Johns Hopkins University Bloomberg School of Public Health, Baltimore, USA; 4 Department of Health Behavior and Nutrition Sciences, University of Delaware, Newark, USA; 5 Department of Epidemiology, University of Pittsburgh School of Public Health, Pittsburgh, USA; 6 Division of Research, Kaiser Permanente Northern California, Pleasanton, USA; 7 Department of Community Health Sciences, University of Illinois Chicago School of Public Health, Chicago, USA; 8 Lifecourse Epidemiology of Adiposity and Diabetes (LEAD) Center, University of Colorado Anschutz Medical Campus, Aurora, USA; 9 Department of Psychology, Emory University, Atlanta, USA; 10 Department of Pediatrics, Icahn School of Medicine at Mount Sinai, New York, USA; 11 Department of Public Health Sciences, MIND Institute, University of California, School of Medicine, Davis, USA; 12 Department of Communication Sciences and Disorders, Northeastern University, Boston, USA

**Keywords:** depression, externalizing and internalizing behaviors, pregnancy, vitamin D

## Abstract

**Background:**

Prenatal depression is associated with offspring behavioral problems, but heterogeneity in the strength of this association is not well understood. Maternal vitamin D concentration during pregnancy is important for fetal brain development and may help explain this variability, with potential differences by timing of exposure and maternal race.

**Methods:**

Using data from 1,451 mother–child pairs in the Environmental influences on Child Health Outcomes cohort, linear mixed-effects models examined associations between prenatal depressive symptom severity, gestational 25-hydroxy-vitamin D (25[OH]D) concentrations, and internalizing and externalizing behaviors in preschool-aged children. Analyses were stratified by common 25(OH)D deficiency thresholds, prenatal timing, and race.

**Results:**

Prenatal depressive symptom severity was associated with greater child internalizing (***β*** = 0.18, 95% CI = 0.11, 0.25) and externalizing (***β*** = 0.21, 95% CI = 0.14, 0.28) behaviors. Gestational 25(OH)D concentration did not moderate depression effect estimates in adjusted models. In stratified analyses, the association between prenatal depressive symptoms and child externalizing behaviors persisted regardless of 25(OH)D threshold levels, but the association with internalizing behaviors attenuated at 25(OH)D < 20 ng/mL. Timing of 25(OH)D measurement (early/late pregnancy) did not modify relationships. Higher gestational 25(OH)D was associated with fewer externalizing problems among offspring of Black mothers only.

**Conclusions:**

Prenatal depressive symptoms showed robust associations with child behavioral problems, largely independent of gestational 25(OH)D. However, attenuated risk for internalizing behaviors with low vitamin D levels warrants investigation of social–environmental factors.

Fetal programming provides a model for understanding the developmental origins of health and disease that focuses on *in utero* exposures prompting adaptive responses that influence future development, behavior, and biology (Barker, 2003; Gluckman et al., 2010). As the most common complication of pregnancy (American College of Obstetricians and Gynecologists, 2018; Bauman et al., 2020), maternal depression is one such condition that has been linked to a range of child developmental outcomes, including psychopathology. Thus, prospective observational studies have shown that prenatal depressive symptomatology is associated with internalizing and externalizing behaviors in offspring even when postpartum depressive symptoms are taken into account, suggesting a unique etiological role of prenatal exposure (Barker et al., 2011; Lahti et al., 2017; O’Donnell et al., 2014). However, there is also considerable variability in the strength of this association. For example, two recent meta-analyses of longitudinal studies revealed significant heterogeneity in estimated effects of prenatal depression on child externalizing behavior after adjusting for many study characteristics (Tung et al., 2024; Tusa et al., 2024). Nonetheless, efforts to understand this heterogeneity remain limited despite the high clinical relevance.

Adequate nutrition during gestation is a critical component of healthy fetal growth and development. Vitamin D, derived from sunlight exposure and dietary intake/supplements, is an essential micronutrient and a neuroactive steroid that plays an important role in fetal brain development (Eyles, 2021; Wagner & Hollis, 2018) and placental function, including inflammation regulation (Calton et al., 2015; Liu et al., 2011). Increasing evidence points to an inverse association between maternal circulating 25-hydroxyvitamin D (25[OH]D) during pregnancy and later internalizing and externalizing behaviors in school-aged offspring (Daraki et al., 2018; Melough et al., 2021; Melough et al., 2023; Morales et al., 2015; Sucksdorff et al., 2021). Additionally, evidence suggests that 25(OH)D concentration in the first and second trimesters may be more critical for offspring neurodevelopment than the concentration in late gestation or at the time of birth (Garcia-Serna & Morales, 2020). This timing-dependent association may reflect critical windows of fetal brain development, as neurogenesis occurs predominantly during early pregnancy when vitamin D receptors are highly expressed on neural tissue (Cui & Eyles, 2022). Placental development and maternal immune system adaptation also occur rapidly during this time and may be particularly sensitive to the immunomodulatory effects of vitamin D (Tamblyn et al., 2022).

Taken together, results suggest that gestational 25(OH)D not only contributes to child neurodevelopmental outcomes but also could attenuate the negative effects of prenatal depression on child neurodevelopment via several biologically plausible pathways. For example, 25(OH)D plays a role in regulating certain neurotransmitters, including serotonin (Patrick & Ames, 2014) and dopamine (Pertile et al., 2016), that are implicated in the pathophysiology of depression (Dunlop & Nemeroff, 2007; Jauhar et al., 2023) and developmental pathways from fetus to child (Oberlander, 2012). Circulating 25(OH)D could also mitigate depression-related alterations to the immune system (e.g. inflammatory cytokine production) that have been linked to child emotional and behavioral problems (Gustafsson et al., 2018). Furthermore, given that vitamin D deficiency is associated with placental dysfunction (Gerovasili et al., 2025), and that alterations in the placental lipidome have been implicated in the association between prenatal depression and offspring socioemotional problems (Wong et al., 2021), higher gestational 25(OH)D concentrations may promote placental health and attenuate fetal exposure to the biological sequelae of maternal prenatal distress. Additionally, gestational 25(OH)D may modulate the programming effects of prenatal distress-related glucocorticoid exposure on fetal neurodevelopment, as vitamin D and glucocorticoid receptors coexist in the hippocampus and prefrontal cortex (Charil et al., 2010). However, despite the theoretical and clinical potential of vitamin D as a moderator of the association between prenatal depression and child behavioral outcomes, research is lacking. One study from a birth cohort in China showed that cord blood 25(OH)D levels above 10 ng/mL attenuated the adverse effects of prenatal depression on offspring risk for attention-deficit/hyperactivity disorder in the preschool period (Ma et al., 2021). Given that 25(OH)D concentrations in prenatal and cord blood samples may not be fully comparable (Keim et al., 2014; Rabbani et al., 2021), additional investigation of this association is clearly warranted.

The strength of attenuation of gestational vitamin D may vary at different concentration thresholds. While optimal 25(OH)D levels for prenatal health remain unclear (Demay et al., 2024; El-Hajj Fuleihan et al., 2015; Marshall et al., 2013), concentrations below 20 ng/mL and below 30 ng/mL have been used to denote deficiency and insufficiency for bone health, respectively (Department of Agriculture and Department of Health and Human Services, 2020). Although the Endocrine Society recently moved away from endorsing specific 25(OH)D thresholds due to a lack of supportive clinical trial evidence (Demay et al., 2024), these thresholds are still commonly used in clinical practice during prenatal health care visits. Thus, probing whether putative moderating effects of gestational vitamin D vary at these thresholds may be critical for developing targeted intervention strategies.

Beyond examining threshold effects, understanding potential variations in these associations by race is also important. Evidence suggests that Black women experience high rates of depression during pregnancy (Bauman et al., 2020; Howard et al., 2014), which often goes undetected in clinical settings (Sidebottom et al., 2023). Circulating levels of 25(OH)D also vary by race (Alzaman et al., 2016), with Black women typically having lower concentrations than White women due to differences in skin pigmentation, dietary patterns, and socioeconomic factors that affect sun exposure, nutrition, and supplement access (Bodnar & Simhan, 2010). Additional evidence suggests that the strength and nature of the associations between prenatal depression and child behavioral outcomes may differ across racial groups. For example, vitamin D receptor gene polymorphisms vary by ancestry (Powe et al., 2013; Reis et al., 2015), which may alter vitamin D metabolism and its neurobiological effects on fetal development (Knabl et al., 2017; Pereira-Santos et al., 2019). Depression symptoms have also been shown to manifest differently across racial and ethnic groups, with Black and African American women exhibiting greater somatic symptoms of depression than White women (Lara-Cinisomo et al., 2020); these differences could influence depression symptom responses to nutritional supplementation or treatment. Furthermore, systemic racial differences in healthcare access and quality (Crear-Perry et al., 2021) may influence both the severity of depression’s effects during pregnancy and the capacity for nutritional factors like vitamin D to buffer against developmental risk. Thus, understanding the extent to which these associations vary by race is critical for tailored interventions that address the full range of biological, social, and healthcare factors shaping maternal and child outcomes.

In the current study, we examined associations between prenatal depression, gestational 25(OH)D, and internalizing and externalizing behaviors in early childhood. Specifically, we hypothesized that gestational 25(OH)D concentration would attenuate the association between prenatal depressive symptom severity and offspring behavioral problems. We also expected that attenuating effects would be stronger among women with 25(OH)D concentrations exceeding two commonly used thresholds (20 and 30 ng/mL) and women for whom gestational 25(OH)D was assessed in early- to mid-pregnancy (<20 weeks gestation) compared with mid- to late pregnancy (≥ 20 weeks gestation). Finally, given known racial differences in both prenatal depression rates and circulating 25(OH)D levels, we explored whether vitamin D modified the association between prenatal depression and child behavioral outcomes in separate analyses for Black women and White women.

## Methods

### Sample

The study sample comprised 1,451 biological mother–child pairs participating in the Environmental influences on Child Health Outcomes (ECHO) Cohort. The ECHO Cohort consists of 69 pregnancy and pediatric cohort sites from across the United States and was established in 2016 to investigate the impact of an array of prenatal exposures on child health and development (Knapp et al., 2023). The study protocol was approved by local and/or single ECHO Institutional Review Boards. Written informed consent or parental/guardian permission was obtained along with child assent, as appropriate, for participation in the ECHO Cohort Data and Biospecimen Collection Protocol and for participation in specific study sites. The current analyses include biological mother–child pairs from five ECHO cohort sites that met inclusion criteria for the study: available gestational 25(OH)D data, singleton births, prenatal maternal depression measures, and maternal reports of internalizing and externalizing behaviors in preschool-aged offspring. For families with multiple children enrolled in the ECHO Cohort, one child was randomly selected for inclusion (see Supplemental Figure 1).

### Measures

#### Gestational 25(OH)D

Concentration of 25(OH)D (ng/mL) was measured from serum or plasma specimens collected during pregnancy. Gestational age at specimen collection ranged from 2 to 35 weeks. If more than one assay result was available in pregnancy, the earliest measure was selected for analysis since prior research indicates an early critical window for the impact of gestational 25(OH)D on fetal brain development (Garcia-Serna & Morales, 2020). Gestational 25(OH)D assay results that were below the lower limit of detection (LLOD) were set to LLOD/√ 2 (Hornung & Reed, 1990). LLOD substitutions were applied to 25(OH)D2 and 25(OH)D3 analytes separately before including them in the total gestational 25(OH)D metric used in analysis. Gestational 25(OH)D levels were modeled as continuous values and scaled to 10 ng/mL increments for regression analyses to aid in interpretation.

#### Prenatal depressive symptoms

Prenatal depressive symptoms were self-reported using the Edinburgh Postnatal Depression Scale (Cox et al., 1987), the Brief Symptom Inventory (Derogatis, 1993), or the Center for Epidemiological Studies Depression Scale (Radloff, 1977). These measures were harmonized to the Patient-Reported Outcomes Measurement Information System (PROMIS) T-score metric using a previously constructed and validated crosswalk table (Blackwell et al., 2021; Kaat et al., 2017). The average PROMIS T-score (normed for the general US population) is 50 (SD = 10), with cutoffs indicating depressive symptom severity: ≤ 55 = normal, > 55 = mild-to-severe range (Kroenke et al., 2020). Depression T-scores were modeled as continuous values for analysis.

#### Child behavioral outcomes

Child behavioral outcomes were measured via maternal report on the Child Behavior Checklist Preschool version for children aged 1.5–5 years (CBCL/1½-5) (Achenbach & Rescorla, 2000). Internalizing and externalizing problem scores were computed from items rated on three-point ordinal scales (0 = Not true, 1 = Somewhat or sometimes true, and 2 = Very or often true). Internalizing problems combined four subscales, that is, emotionally reactive, anxious/depressed, somatic complaints, and withdrawn, while externalizing problems reflected attention problems and aggressive behaviors. Scores were age- and sex-standardized based on a nationally representative sample and transformed to T-scores, with a mean value of 50 and standard deviation of 10 (Achenbach & Rescorla, 2000). If there were multiple preschool-aged CBCL T-scores available for the same child at different ages, the older age was selected for use in analysis. CBCL T-scores were treated as continuous measures in the analysis. T-scores ≥60 and ≤ 63 represent scores in the borderline clinical range, and T-scores ≥64 represent scores in the clinical range.

#### Covariates

Because depression-related outcomes are linked to sociodemographic characteristics (Segre et al., 2007), we included maternal age at delivery, the highest maternal educational attainment at the prenatal assessment (high school degree, general educational development, or did not complete high school; some college or associate’s degree; bachelor’s degree; or post-graduate degree), child sex (male or female), and continuous child age at CBCL administration. These variables were selected because they (i) precede the exposure (prenatal depression) and child behavioral outcomes, (ii) are widely recognized as core demographic confounders in perinatal mental health research, and (iii) were available in harmonized form across cohort sites. Overall, the missingness of each included covariate ranged from 0 to 0.6%.

#### Multiple imputation

Multiple imputations by chained equations were performed for missing covariate data using the ‘mice’ package in R (Van Buuren & Groothuis-Oudshoorn, 2011). All variables of interest, plus additional variables that were informative of missingness, were included in the imputation model (Van Buuren, 2018). Cohort site membership was included as a clustering variable. We imputed 50 datasets with 5 iterations of each imputation. Trace plots and distributions of imputed versus observed data were visually examined. Regression estimates from the imputed datasets were pooled using Rubin’s rules with the mice::pool() function.

#### Statistical analysis

We used linear mixed effect models, including a random intercept for cohort site, to estimate the associations of prenatal depressive symptom scores with child behavior outcomes while adjusting for covariates. The inclusion of the random intercept for cohort site accounted for differences in 25(OH)D assay methods used by each site. Regression analyses were conducted using the ‘lme4’ package in R (Bates et al., 2015). Effect modification was formally tested by adding the product of continuous gestational 25(OH)D concentration and prenatal depression symptom scores. To determine whether there were differential associations by commonly used thresholds, we stratified analyses at 25(OH)D < 20 and ≥ 20 ng/mL and at <30 and ≥ 30 ng/mL, and ran interaction tests of depression by the dichotomized 25(OH)D variables. We displayed the associations of prenatal depressive symptom scores on child outcome by different levels of gestational 25(OH)D in a randomly selected imputed dataset using the ‘interactions’ R package (Long, 2024). Marginal effects of the interactions with 95% CIs were graphed for 25(OH)D < 20 and ≥ 20 ng/mL and for <30 and ≥ 30 ng/mL. We examined whether potential associations varied by timing of 25(OH)D assessment by stratifying models according to whether specimens were collected before 20 weeks of pregnancy or at and after 20 weeks of pregnancy. We also explored gestational 25(OH)D effect modification in models conducted separately for Black and White maternal participants. Finally, we assessed whether associations varied after the removal of individual cohort sites by performing leave-one-out analyses. All analyses were performed using the R statistical software package, version 4.4.0 (R Core Team, 2024). Summary measures and frequencies were calculated using the ‘table1’ package in R (Rich, 2023).

## Results

### Sample characteristics

Descriptive statistics are shown in Table 1 (see Supplemental Table 1 for individual site summaries). Mean age at the time of delivery was 27.3 years (SD = 5.50), and 41.6% of participants were primiparous. The analytic sample differed in racial composition from the overall ECHO cohort: 60.6% of participants identified as Black, 30.9% as White, 1% as Asian, 4.2% as more than one race, and 1.6% another race, including American Indian/Alaska Native American, Native Hawaiian, or Pacific Islander. Most participants identified as non-Hispanic (91.9%). About half of the sample had earned a bachelor’s degree or higher (48.5%).

**Table 1.** Descriptive statistics Table 1. long description.

	Study population
	*N* = 1,451
**Maternal age at delivery (years)**	
Mean (SD)	27.3 (5.50)
Median [Min, Max]	27.0 [16.0, 41.0]
Missing	1 (0.1%)
**Gestational week at delivery**	
Mean (SD)	38.8 (1.58)
Median [Min, Max]	39.0 [23.0, 42.0]
**Preterm birth**	
Term (≥37 weeks)	1,359 (93.7%)
Preterm (<37 weeks)	92 (6.3%)
**Birthweight categories**	
Extremely low or very low birthweight (<1,500 g)	8 (0.5%)
Moderately low birth weight (≥1,500 g and <2,500 g)	78 (5.4%)
Normal birthweight (≥2,500 g and <4,000 g)	1,269 (87.5%)
Grade 1 macrosomia (≥4,000 g and <4500 g)	84 (5.8%)
Grade 2 macrosomia (≥4,500 g and <5,000 g)	12 (0.8%)
**Highest level of maternal education**	
Less than high school	39 (2.7%)
High school degree, GED, or equivalent	308 (21.2%)
Some college, no degree; associate’s degree; trade school	393 (27.1%)
Bachelor’s degree	403 (27.8%)
Master’s degree; professional or doctorate	300 (20.7%)
Missing	8 (0.6%)
**Maternal race**	
American Indian or Alaska Native	5 (0.3%)
Asian	14 (1.0%)
Black	880 (60.6%)
Multiple Races – Black	47 (3.2%)
Multiple Races – not Black	15 (1.0%)
Other	23 (1.6%)
White	449 (30.9%)
Missing	18 (1.2%)
**Maternal ethnicity**	
Non-Hispanic	1,334 (91.9%)
Hispanic	116 (8.0%)
Missing	1 (0.1%)
**Parity**	
0	604 (41.6%)
1	452 (31.2%)
2	225 (15.5%)
> 2	138 (9.5%)
Missing	32 (2.2%)
**Prenatal depression T-score**	
Mean (SD)	45.4 (7.67)
Median [Min, Max]	45.0 [33.0, 74.5]
**Prenatal depression T-score severity**	
None/low (<55)	1,261 (86.9)
Mild (55 to <60)	129 (8.9)
Moderate/severe (≥60)	61 (4.2)
**Prenatal depression instrument**	
EPDS	339 (23.4%)
BSI	1,051 (72.4%)
CESD	61 (4.2%)
**Gestational 25(OH)D concentrations (ng/mL)**	
Mean (SD)	23.8 (10.4)
Median [Min, Max]	22.8 [5.50, 76.1]
**Gestational 25(OH)D status**	
Threshold 1) Concentration < 20 ng/mL	578 (39.8%)
Concentration ≥ 20 ng/mL	873 (60.2%)
Threshold 2) Concentration < 30 ng/mL	1,117 (77.0%)
Concentration ≥ 30 ng/mL	334 (23.0%)
**Gestational age at time of specimen collection, completed weeks**	
Mean (SD)	19.8 (5.62)
Median [Min, Max]	21.0 [2.00, 35.00]
**Gestational age at time of specimen collection**	
<20 weeks gestation	610 (42.0%)
≥20 weeks gestation	841 (58.0%)
**Child sex**	
Male	735 (50.7%)
Female	716 (49.3%)
**Child age at CBCL assessment**	
Mean (SD)	2.86 (0.75)
Median [Min, Max]	3.00 [1.41, 5.33]
**Child race**	
American Indian or Alaska Native	<5
Asian	11 (0.8%)
Black	908 (62.6%)
Multiple Races – Black	46 (3.2%)
Multiple Races – not Black	25 (1.7%)
Native Hawaiian or other Pacific Islander	<5
Other	21 (1.4%)
White	411 (28.3%)
Missing	23 (1.6%)
**Child ethnicity**	
Non-Hispanic	1,240 (85.5%)
Hispanic	138 (9.5%)
Missing	73 (5.0%)
**Child internalizing behavior T-score**	
Mean (SD)	45.9 (10.2)
Median [Min, Max]	45.0 [29.0, 78.0]
**Child internalizing behavior T-score**	
Normal	1,287 (88.7%)
Borderline or clinically relevant score	164 (11.3%)
**Child externalizing behavior T-score**	
Mean (SD)	46.9 (9.71)
Median [Min, Max]	47.0 [28.0, 79.0]
**Child externalizing behavior T-score**	
Normal	1,299 (89.5%)
Borderline or clinically relevant score	152 (10.5%)

*Abbreviations:* 25(OH)D, 25-hydroxy-vitamin D; BSI, Brief Symptom Inventory; CBCL, Child Behavior Checklist; CESD, Center for Epidemiological Studies Depression Scale; EPDS, Edinburgh Postnatal Depression Scale; GED, General Educational Development; SD, standard deviation.

The mean prenatal depressive symptom T-score was 45.4 (SD = 7.67), with 13.1% of participants reporting depressive symptoms in either the mild or moderate-to-severe range. The mean gestational 25(OH)D concentration was 23.8 ng/mL (SD = 10.4); 60.2% of participants had concentrations at or above 20 ng/mL, and 23% had concentrations at or above 30 ng/mL. Across sites, the mean timing of 25(OH)D measurement was 19.8 weeks gestation (42% of samples were collected before 20 weeks; 17.0%, 74.1%, and 8.9% were collected in each respective trimester). The mean 25(OH)D concentration was 24.2 ng/mL (SD = 11.4) before 20 weeks and 23.8 ng/mL (SD = 10.4) at or after 20 weeks. Black participants had lower concentrations of gestational 25(OH)D than White participants (*M* = 20.3 ng/mL, SD = 8.21 vs. *M* = 30 ng/mL, SD = 10.9, respectively, *t* = 16.77, *p* < 0.001). Prenatal depressive symptom severity and gestational 25(OH)D concentration were unrelated (*r* = 0.04, *p* = 0.11).

Child offspring (49.3% female) had a mean age of 2.86 years (SD = 0.75). Mean internalizing and externalizing behavior T-scores were slightly lower than the published CBCL/1½-5 norms (*M* = 45.9, SD = 10.2, and *M* = 46.9, SD = 9.71, respectively); 11.3% and 10.5% of children had internalizing and externalizing T-scores, respectively, in the borderline or clinical range.

### Associations between prenatal depression and child internalizing and externalizing behaviors

In linear mixed-effects models that included cohort site as a random intercept and adjusted for maternal age, maternal highest level of education, child sex, and child age, more severe prenatal depressive symptoms were associated with higher levels of child internalizing and externalizing behaviors (Table 2, Models 1a and 1b).

**Table 2.** Associations between prenatal depressive symptom scores, gestational 25(OH)D concentrations, and child internalizing and externalizing behavior Table 2. long description.

	Internalizing behavior				Externalizing behavior			
	Model 1a		Model 2a		Model 1b		Model 2b	
	*β* (95% CI)	*p*	*β* (95% CI)	*p*	*β* (95% CI)	*p*	*β* (95% CI)	*p*
(Intercept)	37.83(31.97, 43.70)	<0.001	43.56(34.58, 52.54)	<0.001	41.19(35.92, 46.46)	<0.001	42.90(34.69,51.10)	<0.001
Prenatal depressive symptoms	0.18(0.11, 0.25)	<0.001	0.06(−0.11, 0.22)	0.505	0.21(0.14, 0.28)	<0.001	0.18(0.03, 0.34)	0.023
Maternal age at delivery	−0.01(−0.12, 0.09)	0.797	−0.01(−0.11, 0.09)	0.847	−0.05(−0.15, 0.05)	0.340	−0.05(−0.15, 0.05)	0.366
Maternal education								
≤ High school	REF		REF		REF		REF	
Some college	−1.57(−3.02, −0.13)	0.033	−1.49(−2.94, −0.04)	0.044	0.15(−1.23, 1.54)	0.827	0.20(−1.19, 1.59)	0.780
College	−2.98(−4.48, −1.47)	<0.001	−2.86(−4.38, −1.34)	<0.001	−1.34(−2.78, 0.10)	0.067	−1.26(−2.72, 0.20)	0.090
Post-graduate	−4.55(−6.25, −2.85)	<0.001	−4.41(−6.14, −2.69)	<0.001	−2.00(−3.63, −0.37)	0.016	−1.91(−3.56, −0.26)	0.024
Child sex								
Male	REF		REF		REF		REF	
Female	−0.05(−1.07, 0.98)	0.93	−0.05(−1.07, 0.98)	0.927	−1.62(−2.60, −0.64)	0.001	−1.62(−2.60, −0.64)	0.001
Child age at CBCL assessment	0.56(−0.27, 1.39)	0.184	0.53(−0.31, 1.37)	0.216	−0.67(−1.44, 0.10)	0.089	−0.70(−1.47, 0.08)	0.080
Gestational 25(OH)D (per 10 ng/mL)	–	–	−2.63(−5.60, 0.34)	0.082	–	–	−0.77(−3.57, 2.02)	0.587
Prenatal depressive symptoms × 25(OH)D	–	–	0.06(−0.01, 0.12)	0.089	–	–	0.01(−0.05, 0.07)	0.646

*Note:* Beta coefficients are from a linear mixed-effects model with ECHO cohort site membership as the cluster variable.

*Abbreviations: 25(OH)D, 25-hydroxy-vitamin D; CBCL, Child Behavior Checklist; CI, confidence interval.*

### Associations between prenatal depression and child internalizing and externalizing behaviors as a function of 25(OH)D

We examined the extent to which 25(OH)D attenuated associations between prenatal depressive symptom scores and child behavior by including the gestational 25(OH)D main effect and the product of prenatal depressive symptom score and continuous gestational 25(OH)D in the linear mixed-effects models. The interaction terms were nonsignificant in both the adjusted model for child internalizing behavior (*β* = 0.06, *p* = 0.09, Table 2, Model 2a) and the adjusted model for child externalizing behavior (*β* = 0.01, *p* = 0.65, Table 2, Model 2b).

**Figure 1.**
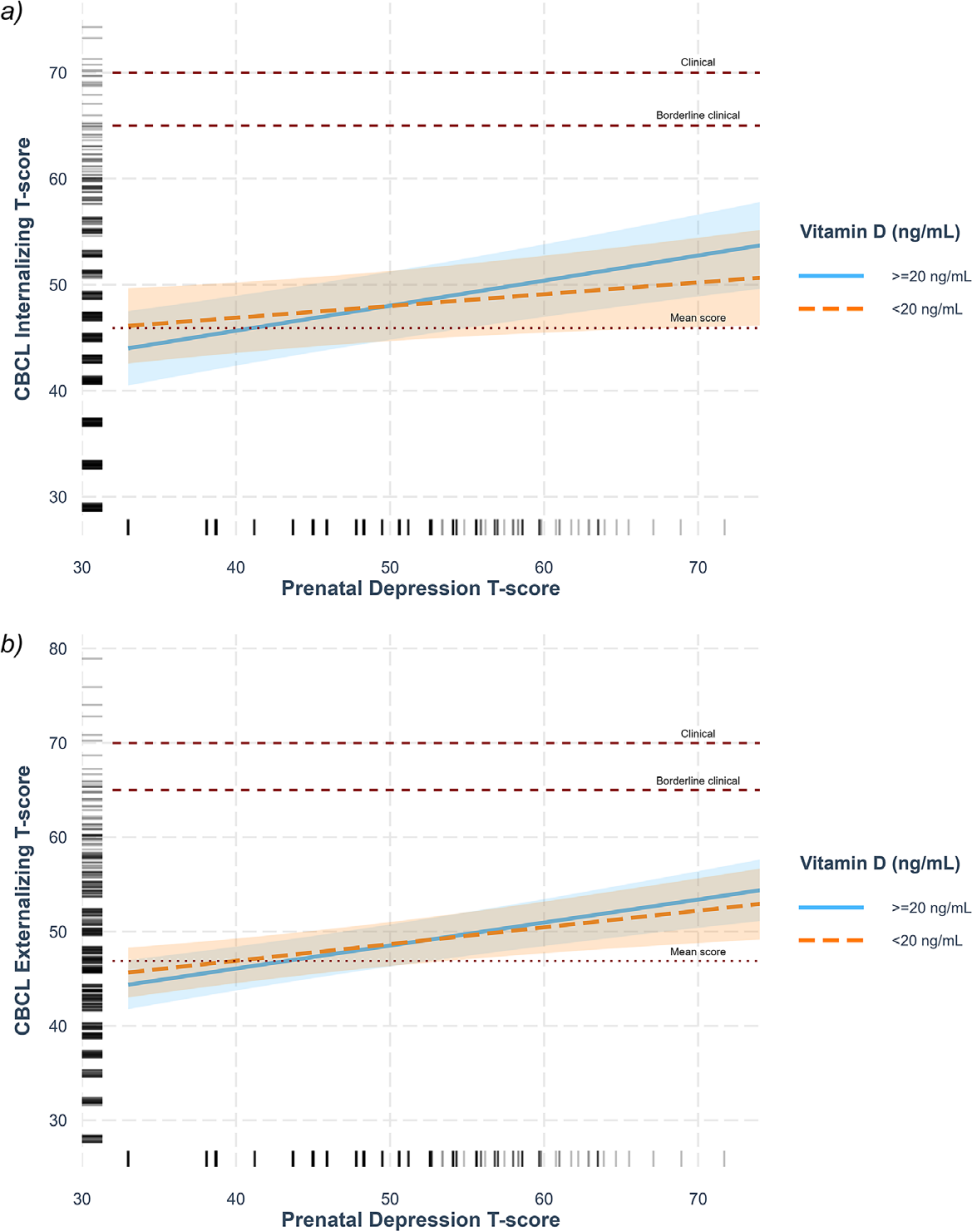
Interaction plots: Prenatal depressive symptoms by vitamin D concentration predicting offspring (a) internalizing behaviors and (b) externalizing behaviors. *Note*: CBCL, Child Behavior Checklist. Figure 1. long description.

Figure 1 illustrates the associations between prenatal depressive symptom severity and child internalizing and externalizing behaviors with regression lines and 95% confidence intervals around gestational 25(OH)D concentrations of <20 and ≥ 20 ng/mL. The largely overlapping confidence intervals in both plots indicate that the associations between prenatal depressive symptoms and child internalizing and externalizing behaviors do not vary as a function of the 25(OH)D concentration.

To aid interpretation of the nonsignificant moderation effect, we additionally examined simple slopes of the association between prenatal depressive symptoms and child behavior at clinically relevant 25(OH)D concentrations (20 and 30 ng/mL). Consistent with the null interaction, all simple slope estimates overlapped substantially (e.g. internalizing slope at 20 ng/mL: *β* = 0.17, 95% CI = 0.09–0.24; at 30 ng/mL: *β* = 0.22, 95% CI = 0.14–0.31 and externalizing slope at 20 ng/mL: *β* = 0.21, 95% CI = 0.14–0.28; at 30 ng/mL: *β* = 0.23, 95% CI = 0.14–0.31). Marginal effects plots with 95% confidence intervals are provided in Supplemental Figure 2 and show no appreciable variation in the depression effect across the observed range of gestational 25(OH)D. Quartile sensitivity analyses to assess potential nonlinear associations were also consistent (Supplemental Table 2). These results reinforce the conclusion that 25(OH)D does not meaningfully alter the association between prenatal depressive symptoms and child behavioral outcomes.

**Figure 2.**
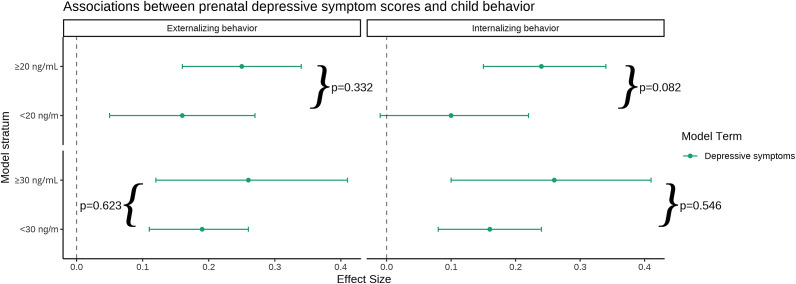
Associations between prenatal depressive symptoms and child internalizing and externalizing behaviors by gestational 25(OH)D strata. Figure 2. long description.

We then stratified by gestational 25(OH)D concentrations to examine whether the association between prenatal depressive symptomatology and preschool behavior varied according to thresholds: < 20 ng/mL vs. ≥ 20 ng/mL and < 30 ng/mL vs. ≥ 30 ng/mL. When concentrations were ≥20 ng/mL, the results showed the same robust associations between prenatal depression and child internalizing and externalizing behaviors in adjusted models as described previously (Supplemental Table 3). When 25(OH)D concentrations were < 20 ng/mL, the association between prenatal depression and child externalizing behaviors remained statistically significant, but the association between prenatal depression and child internalizing behaviors attenuated to non-significance. When probed at the <30 ng/mL vs. ≥ 30 ng/mL threshold, the results remained consistent with the full sample: prenatal depressive symptoms were significantly related to both child internalizing and externalizing behaviors regardless of 25(OH)D stratum (Supplemental Table 4). None of the interaction terms (prenatal depressive symptom scores × 25(OH)D when dichotomized at the 20 or the 30 ng/mL thresholds) were significant (all *p*s > 0.05). Stratum-specific effect estimates are shown in Figure 2.

**Figure 3.**
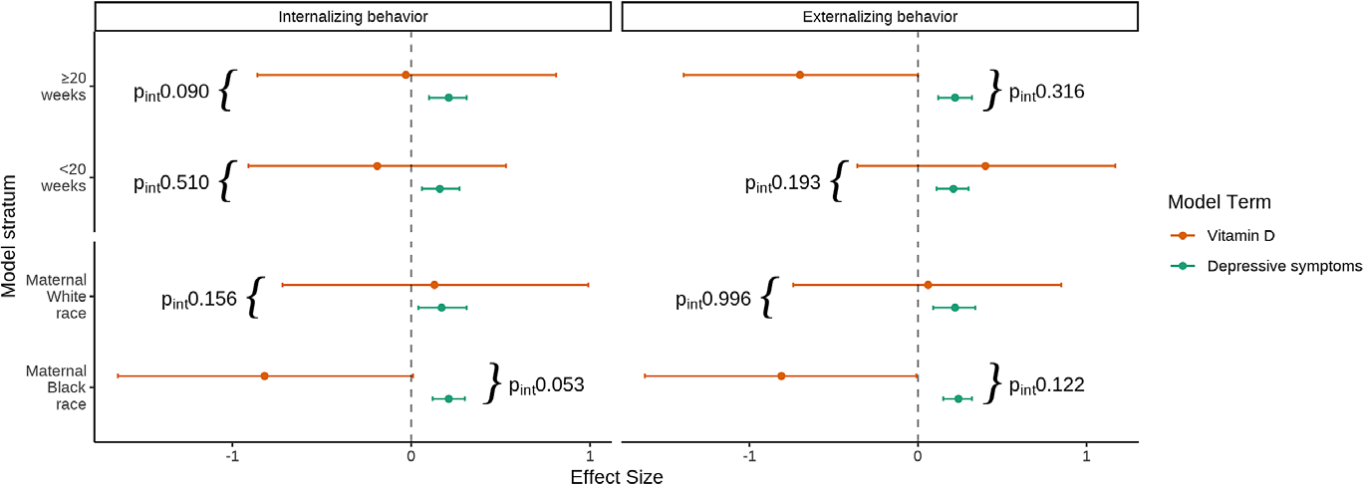
Associations between prenatal vitamin D, depressive symptoms, and child internalizing and externalizing behaviors by timing and maternal race. Figure 3. long description.

We examined whether timing of 25(OH)D measurement modified the associations between prenatal depressive symptoms and child behavior outcomes by stratifying models according to whether blood draws occurred before or after 20 weeks of pregnancy (Supplemental Table 5). The results showed no attenuating effects of 25(OH)D measurement timing on the associations between prenatal depressive symptoms and child outcomes (all *p*s > 0.05, Figure 3).

In race-stratified analyses, we explored the association between prenatal depressive symptoms and 25(OH)D, with child outcomes for Black and White maternal participants. Higher levels of prenatal depressive symptoms were associated with more internalizing and externalizing behaviors in preschool-aged offspring for both racial groups (Supplemental Table 6). Although higher gestational 25(OH)D was associated with fewer externalizing problems among offspring of Black mothers, analyses showed no moderating effect of 25(OH)D concentration on the association between prenatal depression and child behavior outcomes for either Black or White participants (Figure 3).

### Sensitivity analyses

We assessed whether associations varied after the removal of individual cohort sites by performing leave-one-out analyses. Our results were robust, and no single cohort site considerably affected our results (Supplemental Figure 3).

## Discussion

Using data from five US sites in the ECHO Cohort, the current study examined the extent to which 25(OH)D would attenuate the developmental risk conferred by prenatal depression on early childhood behavioral functioning. While our findings confirmed previously reported associations between prenatal depressive symptom severity and higher levels of child internalizing and externalizing problems, they provided no support for our primary hypothesis regarding the protective effects of 25(OH)D, whether measured continuously or probed at commonly used thresholds for health. In addition, no time-specific associations emerged when analyses were stratified by early versus late prenatal 25(OH)D measurement. Collectively, these results suggest that vitamin D is not a key factor in accounting for heterogeneity in associations between prenatal depression and child behavioral outcomes, and that efforts to prevent emotional and behavioral problems in offspring should focus primarily on reducing maternal depressive symptoms during pregnancy.

While stratified analyses demonstrated that associations between prenatal depression and child behavioral problems persisted when maternal 25(OH)D concentration exceeded 20 ng/mL, our findings revealed an unexpected relationship below the 20 ng/mL threshold of 25(OH)D: within this range, the estimated effect of prenatal depression on child internalizing behaviors became smaller and attenuated to non-significance. One explanation is that women with vitamin D deficiency in pregnancy may experience multiple co-occurring stressors (e.g. food insecurity, financial strain, limited opportunities for outdoor leisure time, and limited healthcare access) and nutritional deficiencies that introduce complex confounding patterns, potentially obscuring the association between prenatal depression and child outcomes. Supporting this interpretation, maternal education showed stronger associations with child outcomes in the lower, compared with the higher, 25(OH)D stratum, suggesting a greater influence of socioeconomic factors for these individuals. Alternatively, these results may reflect statistical artifacts such as floor effects, reduced statistical power in the smaller low-vitamin D subsample, or distinct nonlinear dose–response associations reflecting differential mechanistic pathways at very low vitamin D levels.

In analyses exploring whether 25(OH)D moderated the association between depression and child outcome differently by race, the results showed that the interaction was not significant, regardless of race. However, in those models, we did observe racial differences in the direct effect of vitamin D on child outcomes that warrant consideration. Specifically, higher concentrations of gestational 25(OH)D were associated with fewer externalizing behaviors among offspring of Black mothers, with a trend also toward fewer internalizing problems. This pattern was not observed among White mother–child dyads, suggesting the possibility of differential threshold effects whereby beneficial impacts may be disproportionately observed among groups characterized by lower gestational vitamin D concentrations. These findings are consistent with prior research with Black mother–child pairs (Hipwell et al., 2024) demonstrating that higher gestational 25(OH)D concentrations were associated with fewer child behavior problems after adjusting for covariates, including prenatal depression and life stressors. However, this result is in direct contrast with a small study showing that the *lowest* prenatal 25(OH)D quartile was associated with fewer child internalizing problems among Black toddlers (Chawla et al., 2019). Although our findings align with prior studies documenting variations in associations between 25(OH)D blood levels and health outcomes among racial groups (Brown et al., 2018; Burris et al., 2015; Holick, 2013; Michos et al., 2015), further investigation is clearly needed. Importantly, while previous work has established that darker skin pigmentation reduces vitamin D synthesis from sunlight (Institute of Medicine, 2011; Webb et al., 2018), self-identified race serves as an inaccurate proxy for skin pigmentation. Thus, the differences observed here likely reflect social determinants of health associated with both race and low 25(OH)D concentrations (Demay et al., 2024) and further highlight the importance of considering racial, ethnic, and sociocultural factors in prenatal mental health research.

### Strengths and limitations

Several methodological strengths increase confidence in the study findings. First, the use of blood concentrations of vitamin D rather than self-reported dietary intake provides an objective biomarker assessment, reducing measurement error and recall bias. Furthermore, assay results from a subset of 296 ECHO participants who provided two biospecimens during pregnancy indicated that the reliability of 25(OH)D measures was high (*r* = .75, *p* < .001). Second, the large multi-cohort dataset incorporating multimodal measures reduces shared method variance and provides enhanced statistical power. Third, our sample demonstrates good generalizability to community populations, with average prenatal depression T-scores slightly below population means but with substantial representation across the severity spectrum and child behavioral outcomes similar to other community-based samples (Bayer et al., 2025).

Despite these strengths, several limitations should be considered. First, child outcomes may also be influenced by maternal postpartum depressive symptoms, which were not included in the current analysis. However, prior studies demonstrate that prenatal depression uniquely predicts child health and developmental outcomes even after accounting for postpartum depression (e.g. Tung et al., 2024). Consistent with this evidence, and with our focus on testing a fetal programming model in which gestational vitamin D may function as an *in-utero* modifier, we did not adjust for postpartum depression, which may itself be influenced by prenatal depression and thus lie on the causal pathway. Second, child behavior was assessed at a single time point, which may not capture persistent changes predicted by a developmental programming framework. In addition, child behaviors were reported by a single informant, which may introduce informant-related bias and limit understanding of behavioral problems across different contexts. Third, sample characteristics may have impacted our assessment of vitamin D effects. The sample was dominated by one large cohort site with a relatively low mean 25(OH)D concentration, and a large percentage of participants had vitamin D levels deemed ‘sufficient’ by conventional thresholds, potentially limiting our ability to detect non-linear effects at the lowest concentrations. Finally, the serial cross-sectional study design may have introduced systematic bias related to the timing of assessment, as participants more likely to receive early prenatal care may have been enrolled earlier in pregnancy, potentially confounding timing effects with other maternal characteristics.

### Clinical implications and future directions

These findings underscore the critical importance of routine prenatal depression screening and intervention regardless of maternal vitamin D status. The persistent associations between prenatal depression and adverse child outcomes across varying 25(OH)D concentrations suggest that interventions targeting maternal mental health may be most impactful, though comprehensive multifactorial approaches addressing multiple risk factors are warranted. In particular, the nonlinear relationships found at low vitamin D levels require further investigation to understand underlying mechanisms and optimize prevention strategies. Future research should examine sex-specific effects, given the literature suggesting larger prenatal effects for boys, and also probe modifying effects across different stress levels to inform tailored interventions.

## Conclusion

The results of this large-scale investigation provided no evidence that gestational 25(OH)D meaningfully attenuates the association between prenatal depression and early childhood emotional and behavioral problems. Rather, the findings highlight prenatal depression as a persistent risk factor requiring clinical attention and suggest that the role of vitamin D in child behavioral development is complex, particularly at very low concentrations and among racially diverse populations.

## Supporting information

10.1017/S0033291726104917.sm001 Hipwell et al. supplementary material 1 Hipwell et al. supplementary material

10.1017/S0033291726104917.sm002 Hipwell et al. supplementary material 2 Hipwell et al. supplementary material

## Data Availability

Select de-identified data from the ECHO Program are available through NICHD’s Data and Specimen Hub (DASH). Information on study data not available on DASH, such as some Indigenous datasets, can be found on the ECHO study DASH webpage.

## Supplementary material

The supplementary material for this article can be found at http://doi.org/10.1017/S0033291726104917.

## Long descriptions

### Table 1. Long description

The table presents data for a study population of N = 1,451.

- Maternal age at delivery: Mean 27.3 years, S D 5.50, range 16.0 to 41.0.

- Gestational week at delivery: Mean 38.8, S D 1.58. 93.7% were term births (greater than or equal to 37 weeks) and 6.3% were preterm.

- Birthweight: 87.5% were normal birthweight (2,500 to 4,000 grams), with 5.9% categorized as low birthweight and 6.6% as macrosomia.

- Maternal education: The largest groups are Bachelor's degree (27.8%) and some college or associate's degree (27.1%).

- Maternal race and ethnicity: 60.6% Black, 30.9% White, and 8.0% Hispanic.

- Parity: 41.6% had 0 previous births, 31.2% had 1, and 15.5% had 2.

- Prenatal depression: Mean T-score of 45.4. Severity was none or low for 86.9% of the population. Instruments used included B S I (72.4%), E P D S (23.4%), and C E S D (4.2%).

- Gestational 25 O H D concentrations: Mean 23.8 nanograms per milliliter. 39.8% were below 20 nanograms per milliliter and 77.0% were below 30 nanograms per milliliter.

- Child characteristics: 50.7% male and 49.3% female. Mean age at C B C L assessment was 2.86 years.

- Child behavior T-scores: Internalizing behavior mean was 45.9, with 11.3% in the borderline or clinical range. Externalizing behavior mean was 46.9, with 10.5% in the borderline or clinical range.

Navigate back to Table 1..

### Table 2. Long description

The table is divided into two main sections: Internalizing behavior (Models 1a and 2a) and Externalizing behavior (Models 1b and 2b). Each model reports beta coefficients with 95 percent C I and p-values.

Key findings include:

* Intercept: Values range from 37.83 to 43.56 across all models, all with p < 0.001.

* Prenatal depressive symptoms: Significant positive association in Model 1a (beta 0.18, p < 0.001), Model 1b (beta 0.21, p < 0.001), and Model 2b (beta 0.18, p = 0.023). Model 2a was non-significant (p = 0.505).

* Maternal age at delivery: No significant associations (p-values 0.340 to 0.847).

* Maternal education: Using ‘High school or less’ as the reference, ‘Post-graduate’ education showed significant negative associations with behavior scores across all models (e.g., Model 2a beta -4.41, p < 0.001; Model 2b beta -1.91, p = 0.024).

* Child sex: Female sex was significantly associated with lower externalizing behavior (Models 1b and 2b beta -1.62, p = 0.001) but not internalizing behavior.

* Child age at C B C L assessment: No significant associations.

* Gestational 25 O H D (per 10 ng/mL): In Model 2a, beta was -2.63 (p = 0.082); in Model 2b, beta was -0.77 (p = 0.587).

* Interaction (Prenatal depressive symptoms multiplied by 25 O H D): Model 2a beta 0.06 (p = 0.089); Model 2b beta 0.01 (p = 0.646).

Navigate back to Table 2..

### Figure 1. Long description

Two vertically stacked line graphs, labeled a and b. Both share an x-axis titled Prenatal Depression T-score ranging from 30 to 70. A legend to the right indicates two groups: Vitamin D greater than or equal to 20 n g / m L represented by a solid blue line, and Vitamin D less than 20 n g / m L represented by a dashed orange line. Shaded areas around lines represent confidence intervals.

Panel a: The y-axis is C B C L Internalizing T-score from 30 to 80. Both lines show a positive linear increase. The solid blue line has a steeper slope, starting lower at low depression scores and ending higher at high depression scores compared to the dashed orange line. Three horizontal reference lines are present: a dotted line at approximately 46 labeled Mean score, a dashed maroon line at 65 labeled Borderline clinical, and a dashed maroon line at 70 labeled Clinical.

Panel b: The y-axis is C B C L Externalizing T-score from 30 to 80. The trends are similar to panel a, with both Vitamin D groups showing a positive linear correlation between prenatal depression and externalizing behaviors. The solid blue line again shows a slightly steeper upward trajectory than the dashed orange line. The same three horizontal reference lines for Mean score, Borderline clinical, and Clinical are positioned at the same y-values as in panel a.

Navigate back to Figure 1..

### Figure 2. Long description

The forest plot is divided into two main panels. The left panel is titled Externalizing behavior and the right panel is titled Internalizing behavior. The X-axis for both represents Effect Size ranging from 0.0 to 0.4. The Y-axis represents Model stratum divided into two vitamin D threshold comparisons.

In the Externalizing behavior panel

* For the stratum greater than or equal to 20 n g forward slash m L, the effect size is approximately 0.25.

* For the stratum less than 20 n g forward slash m L, the effect size is approximately 0.16. A bracket comparing these two shows p equals 0.332.

* For the stratum greater than or equal to 30 n g forward slash m L, the effect size is approximately 0.26.

* For the stratum less than 30 n g forward slash m L, the effect size is approximately 0.19. A bracket comparing these two shows p equals 0.623.

In the Internalizing behavior panel

* For the stratum greater than or equal to 20 n g forward slash m L, the effect size is approximately 0.24.

* For the stratum less than 20 n g forward slash m L, the effect size is approximately 0.10. A bracket comparing these two shows p equals 0.082.

* For the stratum greater than or equal to 30 n g forward slash m L, the effect size is approximately 0.26.

* For the stratum less than 30 n g forward slash m L, the effect size is approximately 0.16. A bracket comparing these two shows p equals 0.546.

All data points are represented by green dots with horizontal error bars indicating confidence intervals. A vertical dashed line at 0.0 indicates the null effect. A legend on the right identifies the green dot as the Model Term for Depressive symptoms.

Navigate back to Figure 2..

### Figure 3. Long description

The forest plot is divided into two main panels: Internalizing behavior on the left and Externalizing behavior on the right. The X-axis represents Effect Size ranging from negative 1 to 1, with a dashed vertical line at 0 indicating no effect. The Y-axis, labeled Model stratum, is divided into four rows: greater than or equal to 20 weeks, less than 20 weeks, Maternal White race, and Maternal Black race.

Each row contains two data points with horizontal error bars: orange circles for Vitamin D and green circles for Depressive symptoms. Brackets group these pairs with a p sub int value.

In the Internalizing behavior panel:

* Greater than or equal to 20 weeks: Vitamin D is near 0; Depressive symptoms is positive. p sub int 0.090.

* Less than 20 weeks: Vitamin D is negative; Depressive symptoms is positive. p sub int 0.510.

* Maternal White race: Vitamin D is near 0; Depressive symptoms is positive. p sub int 0.156.

* Maternal Black race: Vitamin D is strongly negative; Depressive symptoms is positive. p sub int 0.053.

In the Externalizing behavior panel:

* Greater than or equal to 20 weeks: Vitamin D is negative; Depressive symptoms is positive. p sub int 0.316.

* Less than 20 weeks: Vitamin D is positive; Depressive symptoms is positive. p sub int 0.193.

* Maternal White race: Vitamin D is near 0; Depressive symptoms is positive. p sub int 0.996.

* Maternal Black race: Vitamin D is strongly negative; Depressive symptoms is positive. p sub int 0.122.

Navigate back to Figure 3..
