# Supplementary material for: Does gestational vitamin D attenuate the negative effects of prenatal depression on offspring emotional and behavioral problems? Findings in the ECHO cohort
Source: Psychol Med. 2026 Jun 16;56:e194. doi: 10.1017/S0033291726104917 (PMC13280691; doi:10.1017/S0033291726104917)
Supplement: Hipwell et al. supplementary material 1 — Hipwell et al. supplementary material [file S0033291726104917sup001.docx]

**SUPPLEMENTAL MATERIAL**

**Does Gestational Vitamin D Attenuate the Negative Effects of Prenatal Depression on Offspring Emotional and Behavioral Problems? Findings in the ECHO Cohort**

Alison E. Hipwell, PhD, ClinPsyD* and Irene Tung, PhD*, Meredith Palmore, MHS, Melissa M. Melough, PhD, Lisa M. Bodnar, PhD, Lisa A. Croen, PhD, Ashley V. Hill, DrPH, Traci A. Bekelman, PhD, MPH, Patricia A. Brennan, PhD, Kecia N. Carroll, MD, MPH, Rebecca J. Schmidt, PhD, Emily Zimmerman, PhD, CCC-SLP, and Monica McGrath, ScD, for the ECHO Cohort Consortium^†^

*Joint first authors

^†^ See Appendix for full list of collaborators

Supplemental Table 1. Description of the cohort sites included in the analyses

|  | **Exposures** | | | | | | | | |
| --- | --- | --- | --- | --- | --- | --- | --- | --- | --- |
|  |  |  | **25(OH)D concentration (ng/mL)** | | | | **Prenatal timing (weeks)** | | |
| **Lead investigator** | **Specimen type** | **Sample size** | **Mean (SD)** | | **Range** | | **Mean (SD)** | | **Range** |
| 1. Dunlop | Serum | 229 | 20.1 (8.99) | | 6–44.5 | | 10.9 (3.30) | | 6.00–31.0 |
| C. Duarte | Plasma | 11 | 39.17 (9.6) | | 27.08–61.19 | | 34.1 (1.04) | | 32.0–35.0 |
| Q. Zhao | Plasma | 1051 | 22.84 (9.48) | | 5.5–60.2 | | 22.0 (3.77) | | 2.0–29.0 |
| A. Alshawabkeh | Serum | 61 | 37.87 (10.72) | | 15.5–76.1 | | 16.7 (1.81) | | 11.0–23.0 |
| D. Dabelea | Serum | 99 | 32.55 (10.1) | | 13.5–67.6 | | 16.7 (3.16) | | 11.0–24.0 |
|  | **Outcomes** | | | | | | | | |
|  | **Child age** | **Internalizing score** | | | | **Externalizing score** | | | |
|  | **Mean (SD)** | **Mean (SD)** | | **Range** | | **Mean (SD)** | | **Range** | |
| A. Dunlop | 2.24 (0.5) | 47.57 (10.07) | | 29.0–71.0 | | 48.42 (9.74) | | 28.0–76.0 | |
| C. Duarte | 2.12 (0.15) | 46.09 (11.47) | | 29.0–62.0 | | 44.36 (9.03) | | 32.0–62.0 | |
| Q. Zhao | 2.92 (0.63) | 45.72 (10.22) | | 29.0–78.0 | | 46.65 (9.79) | | 28.0–79.0 | |
| A. Alshawabkeh | 2.21 (0.76) | 41.18 (9.48) | | 29.0–64.0 | | 45.61 (8.03) | | 28.0–68.0 | |
| D. Dabelea | 4.14 (0.35) | 46.92 (9.42) | | 29.0–67.0 | | 46.69 (9.61) | | 28.0–70.0 | |

*Abbreviations:* 25(OH)D, 25-hydroxy-vitamin D; SD, standard deviation.

Supplemental Table 2. Associations between prenatal depressive symptoms by 25(OH)D quartile interactions and child behavior outcomes

|  | **Internalizing behavior** | | **Externalizing behavior** | |
| --- | --- | --- | --- | --- |
|  | ***β* [95% CI]** | ***p*** | ***β* [95% CI]** | ***p*** |
| (Intercept) | 41.36(33.61,49.11) | < 0.001 | 43.62(36.71,50.53) | <0.001 |
| Prenatal depressive symptoms | 0.10(-0.04,0.24) | 0.149 | 0.17(0.04,0.30) | 0.011 |
| 25(OH)D Q1 | REF |  | REF |  |
| 25(OH)D Q2 | -6.71(-15.46,2.03) | 0.132 | -4.59(-12.98,3.79) | 0.283 |
| 25(OH)D Q3 | -3.21(-12.08,5.66) | 0.478 | -0.31(-8.81,8.18) | 0.943 |
| 25(OH)D Q4 | -3.89(-12.81,5.03) | 0.393 | 0.06(-8.09,8.22) | 0.988 |
| Maternal age at delivery | -0.01(-0.11,0.09) | 0.855 | -0.05(-0.14,0.05) | 0.369 |
| Maternal education |  |  |  |  |
| ≤ High school | REF |  | REF |  |
| Some college | -1.55(-3.00,-0.10) | 0.036 | 0.19(-1.20,1.57) | 0.792 |
| College | -2.97(-4.50,-1.45) | <0.001 | -1.20(-2.65,0.25) | 0.105 |
| Post-graduate | -4.59(-6.32,-2.86) | <0.001 | -1.85(-3.50,-0.21) | 0.027 |
| Child sex |  |  |  |  |
| Male | REF |  | REF |  |
| Female | -0.07(-1.09,0.95) | 0.892 | -1.64(-2.62,-0.66) | 0.001 |
| Child age at CBCL administration | 0.58(-0.25,1.40) | 0.171 | -0.43(-1.09,0.24) | 0.207 |
| Depression T-score x 25(OH)D Q1 | REF |  | REF |  |
| Depression T-score x 25(OH)D Q2 | 0.16(-0.03,0.35) | 0.100 | 0.10(-0.08,0.29) | 0.267 |
| Depression T-score x 25(OH)D Q3 | 0.06(-0.13,0.26) | 0.512 | -0.02(-0.20,0.17) | 0.862 |
| Depression T-score x 25(OH)D Q4 | 0.08(-0.11,0.28) | 0.393 | -0.02(-0.19,0.16) | 0.846 |

*Note:* ANOVA p-value for improvement of fit with interaction terms: p=0.433 (internalizing behavior model); p=0.509 (externalizing behavior model)

*Abbreviations:* 25(OH)D, 25-hydroxy-vitamin D; CBCL, Child Behavior Checklist; CI, confidence interval.

Supplemental Table 3. Associations between prenatal depressive symptoms and child behavior outcomes stratified by prenatal 25(OH)D levels (< 20, ≥ 20ng/ml).

|  | **Internalizing Behavior** | | | | **Externalizing Behavior** | | | |
| --- | --- | --- | --- | --- | --- | --- | --- | --- |
|  | **25(OH)D levels < 20 ng/ml**  (n=578) | | **25(OH)D levels > 20 ng/ml**  (n=873) | | **25(OH)D levels < 20 ng/ml**  (n=578) | | **25(OH)D levels > 20 ng/ml**  (n=873) | |
|  | ***β* [95% CI]^a^** | ***p*** | ***β* [95% CI]^a^** | ***p*** | ***β* [95% CI]^a^** | ***p*** | ***β* [95% CI]^a^** | ***p*** |
| (Intercept) | 45.41(37.14,53.68) | < 0.001 | 33.27(26.05,40.48) | < 0.001 | 47.13(39.02,55.24) | < 0.001 | 37.68(31.26,44.10) | < 0.001 |
| Prenatal depressive symptoms | 0.10(-0.01,0.22) | 0.076 | 0.24(0.15,0.34) | < 0.001 | 0.16(0.05,0.27) | 0.004 | 0.25(0.16,0.34) | < 0.001 |
| Maternal age at delivery | -0.05(-0.22,0.13) | 0.6 | 0.01(-0.12,0.14) | 0.919 | -0.11(-0.28,0.07) | 0.227 | -0.01(-0.13,0.11) | 0.888 |
| Maternal education |  |  |  |  |  |  |  |  |
| ≤ High school | REF |  | REF |  | REF |  | REF |  |
| Some college | -2.09(-4.19,0.01) | 0.051 | -0.82(-2.87,1.23) | 0.435 | 0.15(-1.91,2.21) | 0.886 | 0.22(-1.72,2.15) | 0.827 |
| College | -3.25(-5.64,-0.86) | 0.008 | -2.26(-4.29,-0.23) | 0.029 | -0.82(-3.17,1.52) | 0.492 | -1.46(-3.37,0.45) | 0.134 |
| Post-graduate | -5.78(-8.69,-2.86) | < 0.001 | -3.46(-5.68,-1.24) | 0.002 | -2.03(-4.89,0.83) | 0.163 | -1.89(-3.97,0.20) | 0.077 |
| Child sex |  |  |  |  |  |  |  |  |
| Male | REF |  | REF |  | REF |  | REF |  |
| Female | -0.16(-1.86,1.54) | 0.856 | 0.00(-1.28,1.29) | 0.994 | -1.81(-3.48,-0.14) | 0.034 | -1.49(-2.70,-0.28) | 0.016 |
| Child age at CBCL  administration | 0.11(-1.09,1.31) | 0.862 | 0.67(-0.36,1.69) | 0.202 | -1.02(-2.19,0.16) | 0.09 | -0.49(-1.42,0.45) | 0.306 |

*Note:* *^a^*Beta coefficients are from a linear mixed effects model with ECHO cohort site membership as the cluster variable.

*Abbreviations:* 25(OH)D, 25-hydroxy-vitamin D; CBCL, Child Behavior Checklist; CI, confidence interval.

Supplemental Table 4. Associations between prenatal depressive symptoms and child behavior outcomes stratified by prenatal 25(OH)D levels (<30, ≥ 30ng/ml).

|  | **Internalizing Behavior** | | | | **Externalizing Behavior** | | | |
| --- | --- | --- | --- | --- | --- | --- | --- | --- |
|  | **25(OH)D levels < 30 ng/ml**  (n=1,117) | | **25(OH)D levels > 30 ng/ml**  (n=334) | | **25(OH)D levels < 30 ng/ml**  (n=1,117) | | **25(OH)D levels > 30 ng/ml**  (n=334) | |
|  | ***β* [95% CI]^a^** | ***p*** | ***β* [95% CI]^a^** | ***p*** | ***β* [95% CI]^a^** | ***p*** | ***β* [95% CI]^a^** | ***p*** |
| (Intercept) | 41.14(35.47,46.80) | < 0.001 | 31.62(20.40,42.85) | < 0.001 | 44.19(38.81,49.57) | < 0.001 | 36.97(26.21,47.73) | < 0.001 |
| Prenatal depressive symptoms | 0.16(0.08,0.24) | < 0.001 | 0.26(0.10,0.41) | 0.001 | 0.19(0.11,0.26) | < 0.001 | 0.26(0.12,0.41) | 0.001 |
| Maternal age at delivery | -0.01(-0.13,0.11) | 0.891 | -0.04(-0.24,0.17) | 0.728 | -0.08(-0.19,0.04) | 0.184 | 0.09(-0.11,0.29) | 0.387 |
| Maternal education |  |  |  |  |  |  |  |  |
| ≤ High school | REF |  | REF |  | REF |  | REF |  |
| Some college | -1.58(-3.19,0.04) | 0.055 | -1.38(-5.02,2.27) | 0.458 | 0.87(-0.66,2.40) | 0.267 | -4.14(-7.69,-0.58) | 0.023 |
| College | -3.00(-4.67,-1.34) | < 0.001 | -2.59(-6.41,1.23) | 0.184 | -0.68(-2.26,0.90) | 0.397 | -5.54(-9.27,-1.81) | 0.004 |
| Post-graduate | -5.23(-7.16,-3.31) | < 0.001 | -2.86(-6.86,1.14) | 0.161 | -1.66(-3.50,0.17) | 0.075 | -5.52(-9.42,-1.62) | 0.006 |
| Child sex |  |  |  |  |  |  |  |  |
| Male | REF |  | REF |  | REF |  | REF |  |
| Female | -0.46(-1.65,0.73) | 0.45 | 1.12(-0.90,3.15) | 0.277 | -1.77(-2.90,-0.64) | 0.002 | -1.04(-3.02,0.94) | 0.301 |
| Child age at CBCL administration | 0.25(-0.59,1.08) | 0.567 | 1.22(-0.35,2.79) | 0.127 | -0.87(-1.67,-0.07) | 0.033 | -0.25(-1.74,1.24) | 0.743 |

*Note:* *^a^*Beta coefficients are from a linear mixed effects model with ECHO cohort site membership as the cluster variable.

*Abbreviations:* 25(OH)D, 25-hydroxy-vitamin D; CBCL, Child Behavior Checklist; CI, confidence interval.

|  | **Internalizing Behavior** | | | | **Externalizing Behavior** | | | |
| --- | --- | --- | --- | --- | --- | --- | --- | --- |
|  | **25(OH)D assessment <20 weeks** (n=610) | | **25(OH)D assessment ≥ 20 weeks** (n=841) | | **25(OH)D assessment < 20 weeks** (n=610) | | **25(OH)D assessment ≥ 20 weeks** (n=841) | |
|  | ***β* [95% CI]^a^** | ***p*** | ***β* [95% CI]^a^** | ***p*** | ***β* [95% CI]^a^** | ***p*** | ***β* [95% CI]^a^** | ***p*** |
| (Intercept) | 37.23(28.30,46.16) | < 0.001 | 39.29(32.29,46.28) | < 0.001 | 39.62(31.95,47.28) | < 0.001 | 43.38(36.59,50.17) | < 0.001 |
| Prenatal depressive symptoms | 0.21(0.10,0.31) | < 0.001 | 0.16(0.06,0.27) | 0.003 | 0.21(0.11,0.30) | < 0.001 | 0.22(0.12,0.32) | < 0.001 |
| 25(OH)D (per 10 ng/mL) | -0.03(-0.86,0.81) | 0.952 | -0.19(-0.91,0.53) | 0.609 | 0.40(-0.36,1.17) | 0.302 | -0.70(-1.39,0.00) | 0.051 |
| Maternal age at delivery | -0.03(-0.20,0.14) | 0.737 | 0.00(-0.13,0.14) | 0.978 | -0.05(-0.21,0.11) | 0.54 | -0.03(-0.16,0.10) | 0.658 |
| Maternal education |  |  |  |  |  |  |  |  |
| ≤ High school | REF |  | REF |  | REF |  | REF |  |
| Some college | -2.52(-4.69,-0.34) | 0.023 | -0.63(-2.60,1.34) | 0.531 | -1.27(-3.30,0.77) | 0.222 | 1.47(-0.44,3.38) | 0.132 |
| College | -3.53(-5.96,-1.11) | 0.004 | -2.36(-4.34,-0.38) | 0.019 | -3.34(-5.61,-1.07) | 0.004 | 0.21(-1.71,2.13) | 0.826 |
| Post-graduate | -4.97(-7.71,-2.22) | < 0.001 | -4.02(-6.25,-1.79) | < 0.001 | -2.86(-5.43,-0.29) | 0.029 | -1.12(-3.28,1.04) | 0.309 |
| Child sex |  |  |  |  |  |  |  |  |
| Male | REF |  | REF |  | REF |  | REF |  |
| Female | 0.01(-1.59,1.61) | 0.989 | -0.13(-1.47,1.21) | 0.85 | -1.35(-2.85,0.15) | 0.077 | -1.81(-3.11,-0.51) | 0.006 |
| Child age at CBCL administration | 0.056 (-0.82,1.94) | 0.423 | 0.52(-0.50,1.54) | 0.319 | 0.03(-1.15,1.21) | 0.962 | -1.13(-2.12,-0.14) | 0.026 |

Supplemental Table 5. Associations between prenatal depressive symptoms and child behavior outcomes stratified by timing of 25(OH)D assessment

*Note:* *^a^* Beta coefficients are from a linear mixed effects model with ECHO cohort site membership as the cluster variable.

*Abbreviations:* 25(OH)D, 25-hydroxy-vitamin D; CBCL, Child Behavior Checklist; CI, confidence interval.

Supplemental Table 6. Associations between prenatal depressive symptoms, 25(OH)D, and child behavior outcomes stratified by maternal-reported race

*Note:* *^a^*Beta coefficients are from a linear mixed effects model with ECHO cohort site membership as the cluster variable.

*Abbreviations:* 25(OH)D, 25-hydroxy-vitamin D; CBCL, Child Behavior Checklist; CI, confidence interval.

|  | **White Race** (n=464) | | | | **Black Race** (n=895) | | | |
| --- | --- | --- | --- | --- | --- | --- | --- | --- |
|  | **Internalizing behavior** | | **Externalizing behavior** | | **Internalizing behavior** | | **Externalizing behavior** | |
|  | ***β* [95% CI]** | ***p*** | ***β* [95% CI]** | ***p*** | ***β* [95% CI]** | ***p*** | ***β* [95% CI]** | ***p*** |
| (Intercept) | 40.91(29.76,52.07) | < 0.001 | 41.43(31.23,51.63) | < 0.001 | 39.69(33.28,46.10) | < 0.001 | 43.82(37.58,50.06) | < 0.001 |
| Prenatal depressive symptoms | 0.17(0.04,0.31) | 0.011 | 0.22(0.09,0.34) | 0.001 | 0.21(0.12,0.30) | < 0.001 | 0.24(0.15,0.32) | < 0.001 |
| 25(OH)D (per 10 ng/mL) | 0.13(-0.72,0.99) | 0.756 | 0.06(-0.74,0.85) | 0.887 | -0.82(-1.64,0.01) | 0.053 | -0.81(-1.62,-0.01) | 0.047 |
| Maternal age at delivery | -0.19(-0.39,0.00) | 0.054 | -0.09(-0.27,0.10) | 0.354 | -0.01(-0.14,0.12) | 0.861 | -0.09(-0.22,0.04) | 0.187 |
| Maternal education |  |  |  |  |  |  |  |  |
| ≤ High school | REF |  | REF |  | REF |  | REF |  |
| Some college | -0.54(-4.01,2.92) | 0.759 | 0.97(-2.27,4.21) | 0.557 | -1.92(-3.59,-0.26) | 0.023 | -0.06(-1.68,1.56) | 0.942 |
| College | -2.99(-6.39,0.41) | 0.084 | -2.63(-5.81,0.56) | 0.106 | -2.83(-4.66,-1.00) | 0.003 | -1.11(-2.90,0.67) | 0.222 |
| Post-graduate | -4.94(-8.43,-1.45) | 0.006 | -3.64(-6.91,-0.38) | 0.029 | -5.02(-7.30,-2.74) | <0.001 | -2.02(-4.24,0.21) | 0.075 |
| Child sex |  |  |  |  |  |  |  |  |
| Male | REF |  | REF |  | REF |  | REF |  |
| Female | -0.11(-1.87,1.64) | 0.898 | -2.12(-3.76,-0.48) | 0.012 | -0.41(-1.72,0.91) | 0.544 | -1.60(-2.88,-0.32) | 0.015 |
| Child age at CBCL  administration | 1.34(-0.15,2.83) | 0.077 | 0.11(-1.25,1.47) | 0.873 | 0.34(-0.61,1.29) | 0.481 | -0.92(-1.84,-0.00) | 0.049 |

Supplemental Figure 1. Consort diagram


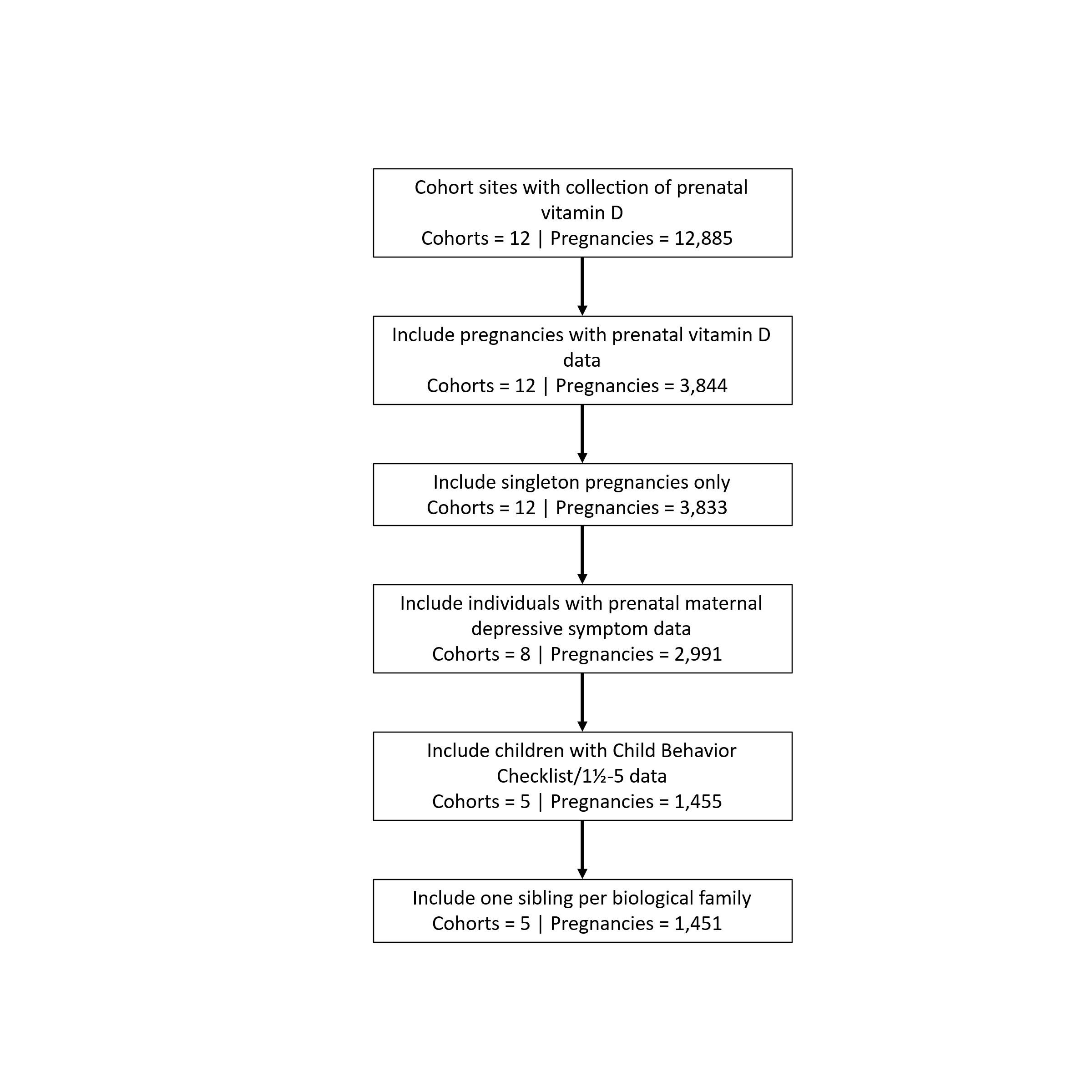


Supplemental Figure 2. Marginal effects of the interactions between prenatal depressive symptoms and 25(OH)D with 95% CIs graphed for < 20ng/mL, ≥ 20ng/mL, < 30ng/mL and ≥ 30ng/mL


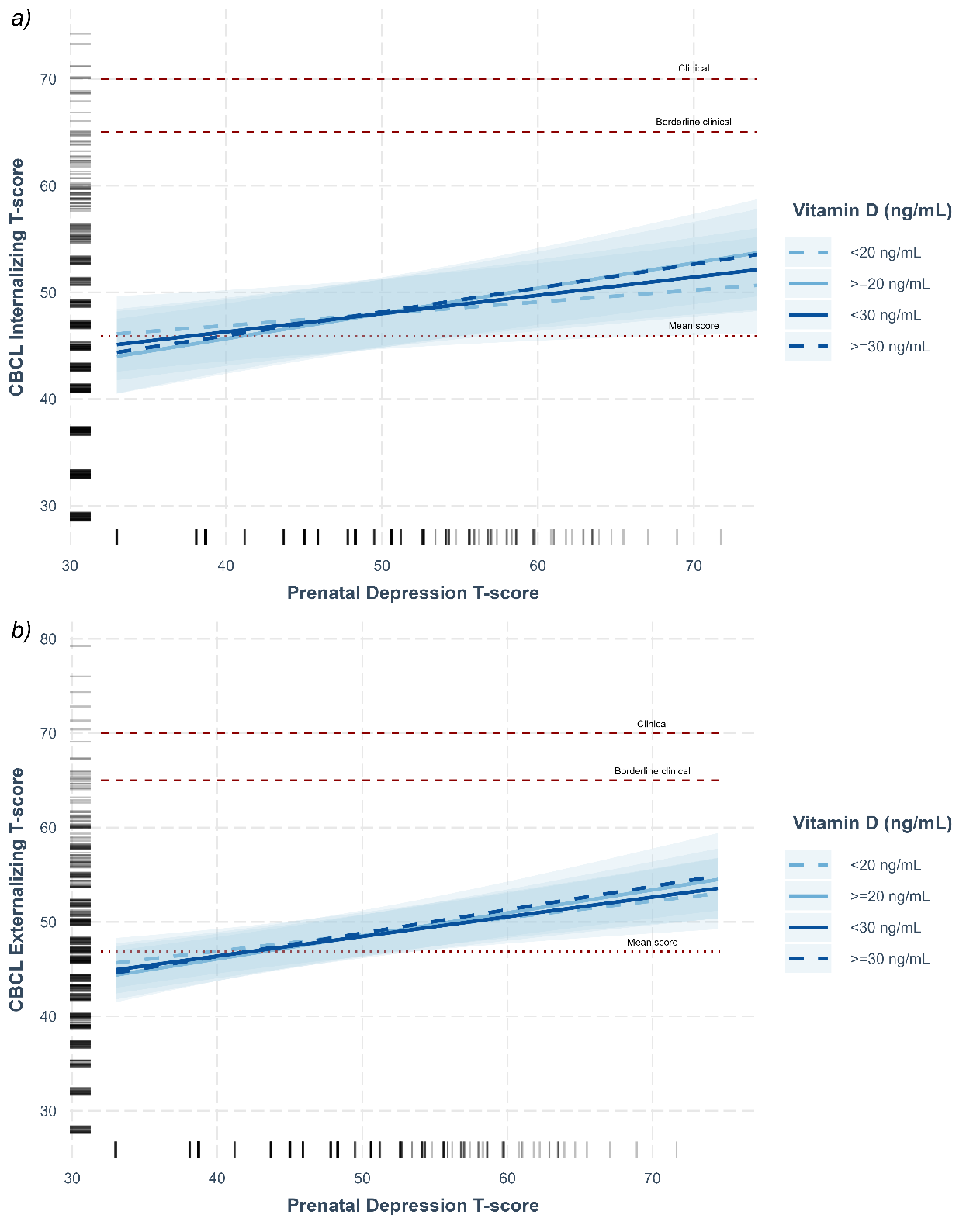


Supplemental Figure 3. Site-adjusted effects of prenatal depressive symptoms on child behavior


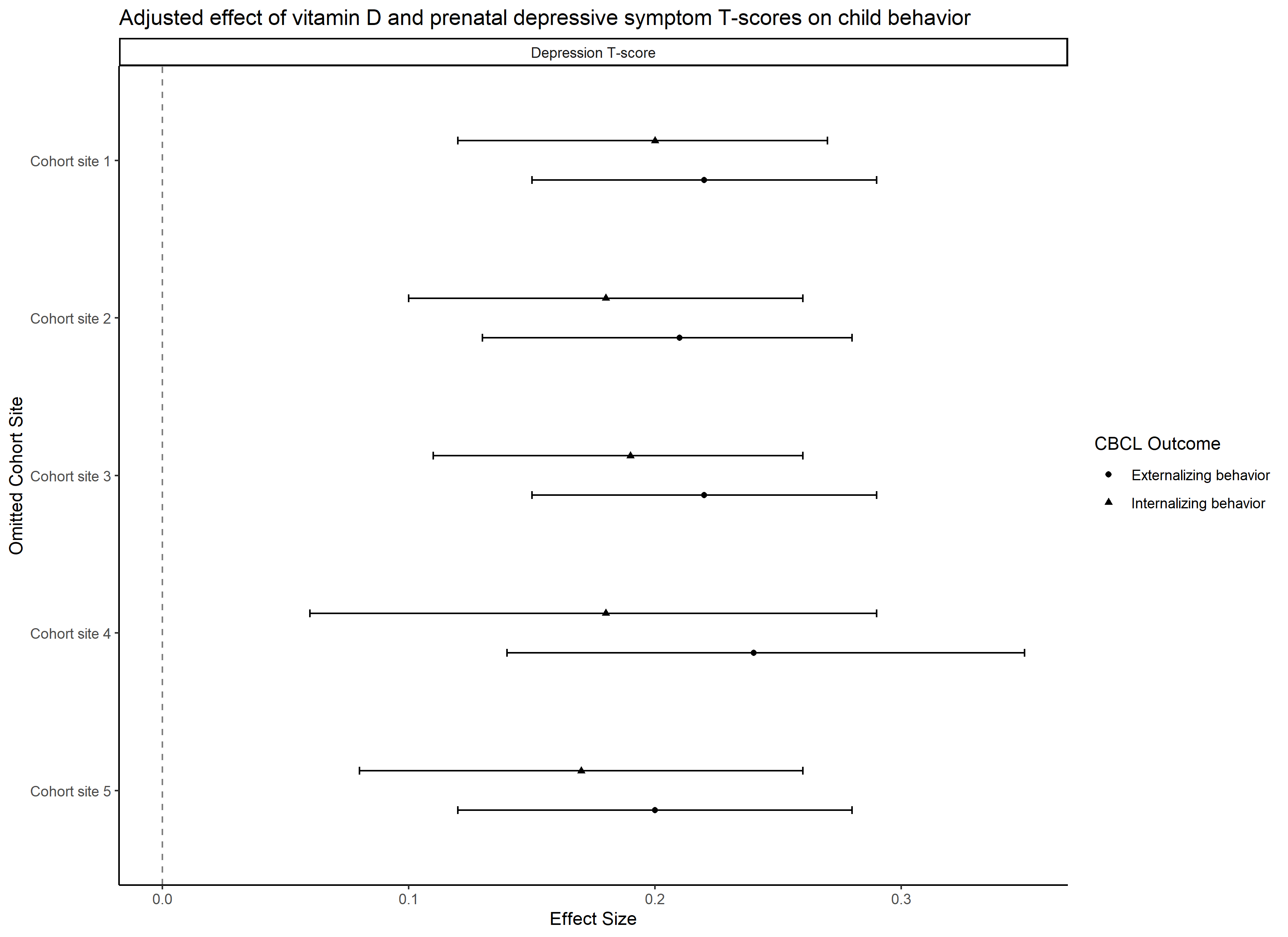


*Abbreviations:* CBCL, Child Behavior Checklist.
